# Histone deacetylase activity mediates thermal plasticity in zebrafish (*Danio rerio*)

**DOI:** 10.1038/s41598-019-44726-x

**Published:** 2019-06-03

**Authors:** Frank Seebacher, Alec I. M. Simmonds

**Affiliations:** School of Life and Environmental Sciences, The University of Sydney, Sydney, NSW 2006 Australia

**Keywords:** Ecophysiology, Animal physiology

## Abstract

Regulatory mechanisms underlying thermal plasticity determine its evolution and potential to confer resilience to climate change. Here we show that class I and II histone deacetylases (HDAC) mediated thermal plasticity globally by shifting metabolomic profiles of cold acclimated zebrafish (*Danio rerio*) away from warm acclimated animals. HDAC activity promoted swimming performance, but reduced slow and fast myosin heavy chain content in cardiac and skeletal muscle. HDAC increased sarco-endoplasmic reticulum ATPase activity in cold-acclimated fish but not in warm-acclimated animals, and it promoted cardiac function (heart rate and relative stroke volume) in cold but not in warm-acclimated animals. HDAC are an evolutionarily ancient group of proteins, and our data show that they mediate the capacity for thermal plasticity, although the actual manifestation of plasticity is likely to be determined by interactions with other regulators such as AMP-activated protein kinase and thyroid hormone.

## Introduction

Resilience of animals to environmental variation determines population persistence, particularly in the current era of rapid climate change^1,2^. Variation in environmental parameters such as temperature can impact animal populations and influence evolutionary responses^3^. Animals can compensate for environmental variation within their lifetime by remodeling their physiology, and thereby alter thermal sensitivities of physiological rates^4,5^. The resulting reversible phenotypic plasticity, or acclimation, benefits performance in variable thermal environments^6–8^. However, the capacity for acclimation differs between phylogenetic groups^8^. To predict the efficacy of acclimation in buffering animals against negative consequences of climate variation therefore requires understanding of the underlying enabling mechanisms and their evolutionary history.

Histone (de)acetylation is an evolutionarily ancient mechanism^9^ that could potentially confer the capacity for acclimation on most organisms. Class I and II histone deacetylases (HDAC) and histone acetyl transferases (HAT) remove or add acetyl groups to nucleosomal histone molecules and thereby repress or promote gene expression, respectively^10^. Acetylation of histones is reversible within organisms, and faster acting than DNA and histone methylation^11,12^, and could represent a mechanism that regulates reversible acclimation of gene expression and physiological functions in response to environmental signals^13,14^. In addition, HDACs can also mediate acetylation of individual protein species and thereby change protein activity post-translationally^15^.

Experimental manipulation of HDAC I and II activity with trichostatin A (TSA) has shown that the interaction between histone acetylation and deacetylation modulates cardiac and skeletal muscle function^16–18^. Additionally, HDAC can form corepressor complexes that inhibit activity of proteins directly^19^. For example, HDAC4 modulates muscle fibre type-specific gene expression programs via its effect on the transcriptional regulator myocyte enhancer factor 2 (MEF2)^17,18,20^. Interestingly, HDAC interact with the AMP-activated protein kinase (AMPK)^21,22^. AMPK acts as an energy sensing molecule that restores cellular energy balance following environmental perturbations^22^. A relative increase in AMP (i.e. a decrease in the ATP:AMP ratio), resulting from a temperature-induced slowing of metabolic reaction rates, for example^23,24^, leads to activation (phosphorylation) of AMPK to restore energy balance^22,23^. Active AMPK (*p*AMPK) exports HDAC4 from the nucleus to the cytosol thereby increasing histone acetylation and altering gene expression programs^9^. In muscle cells, removal of HDAC4 from the nucleus increases expression and activity of MEF2^18^, which promotes expression of slow muscle fibres and therefore more oxidative phenotypes with higher fatigue resistance^25^, among a range of other functions^26,27^. The AMPK-HDAC axis could therefore mediate responses to cold exposure such as increased locomotor performance and cardiac function associated with thermal acclimation^28,29^.

The aim of this study was to determine whether class I and II HDAC activity mediates thermal plasticity in response to temperature change in zebrafish (*Danio rerio*). Zebrafish inhabit a broad thermal niche in their natural environment^30^, and acclimate well to different temperature regimes. Acclimation to cold led to increases in energy metabolism, muscle function and locomotion, and cardiac function^28,29,31^. AMPK-HDAC signaling can potentially affect all of these responses, and we tested the hypothesis that inhibition of class I and II HDACs with TSA^17,18^, which would mimic its removal from the nucleus, induces cold-acclimation responses (Supplementary Fig. S1). As a corollary, we expected AMPK activity (*p*AMPK:AMPK) to decrease with TSA treatment as a result of feedback from reduced HDAC activity, which would restore energy balance by promoting more oxidative phenotypes. Specifically, we predicted that inhibition of HDAC leads to increases in sustained locomotor performance and cardiac function^16,29,31,32^ in warm-acclimated animals. We further predicted that these changes are accompanied by an increase in myosin heavy chain content, and a shift from fast to slow myosin heavy chain (MHC) isoforms in skeletal and heart muscle^33,34^. We tested these hypotheses by conducting a fully factorial experiment with acclimation temperature (three week acclimation to either 18 °C or 28 °C), acute test temperature (18 °C and 28 °C), and TSA treatment (control, DMSO only, TSA dissolved in DMSO) as factors.

## Results

### HDAC altered metabolite profiles differently in warm and cold acclimation

**Figure 1:**
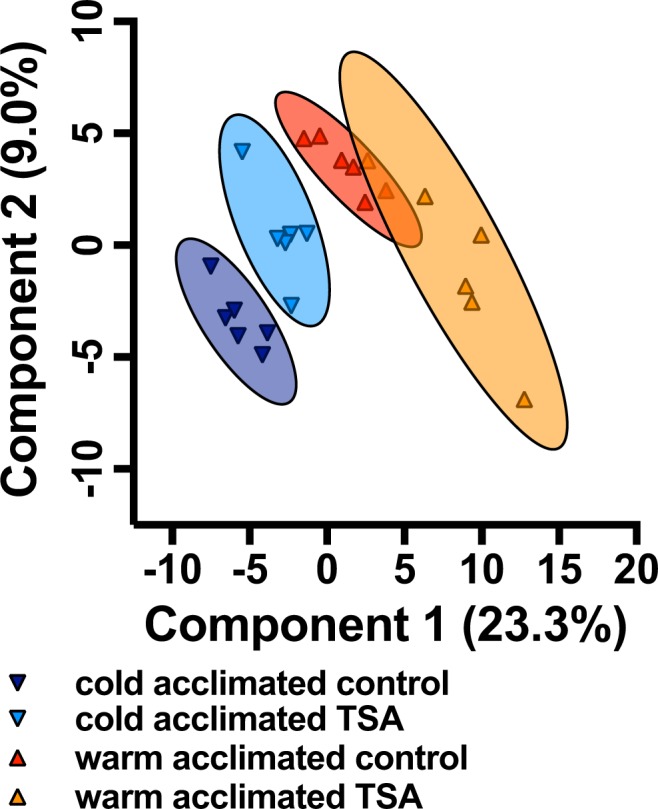
Results from the metabolomics analysis. The first two components from the partial least squares discriminant analysis show that metabolite profiles from cold (18 °C) acclimated TSA-treated fish were significantly different from cold acclimated control fish (95% confidence intervals indicated by shaded ovals) and shifted towards warm-acclimated control fish. However, both cold acclimated groups were significantly different from the warm-acclimated fish, and 95% confidence intervals overlapped between warm-acclimated control and TSA treated fish. Symbols indicate individual samples (N = 6 fish per treatment).

In the multi-group comparison, the metabolite profile of cold-acclimated control fish was significantly different from all the other groups (non-overlap of 95% confidence intervals; Fig. 1). TSA shifted the metabolomics profile of the cold acclimated fish towards the warm-acclimated fish, but cold-acclimated TSA-treated fish were significantly different from all other groups. The 95% confidence intervals overlapped between warm-acclimated control and TSA-treated fish indicating that these groups are similar statistically (Fig. 1).

Pairwise comparisons showed that there were 41 compounds differently regulated between cold acclimated control and TSA-treated fish, 172 compounds between control cold and warm acclimated fish, 77 compounds between cold acclimated TSA-treated fish and warm acclimated control fish, and 46 compounds between warm acclimated control and TSA-treated fish (see also Metabolomics compounds in Supplementary Material).

### HDAC modified acute thermal sensitivity of swimming performance

**Figure 2:**
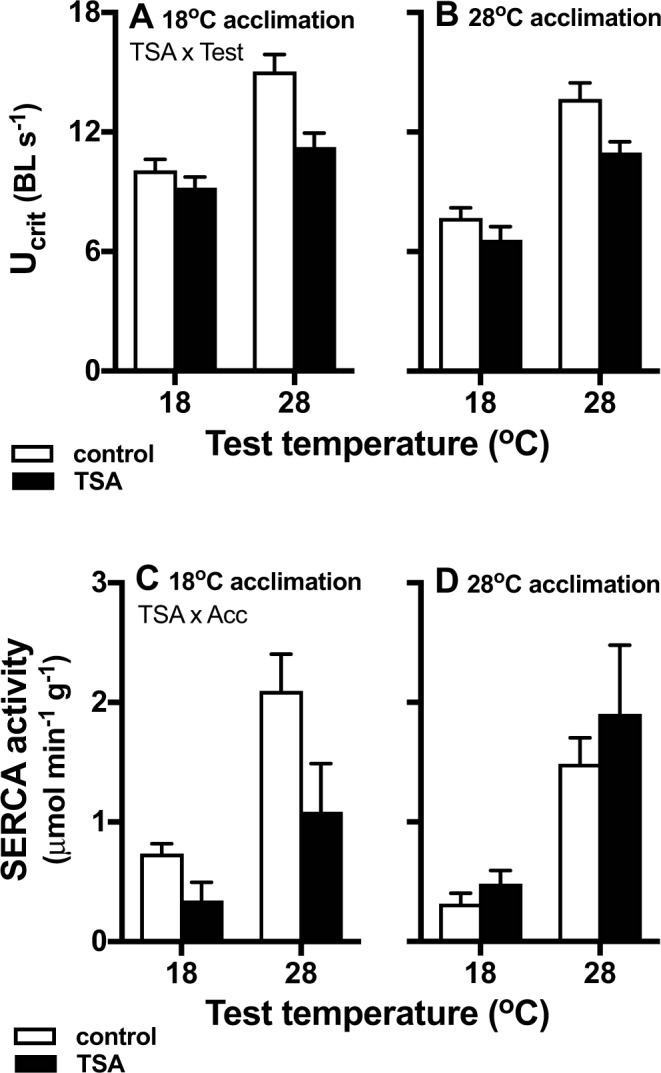
Effect of trichostatin A (TSA) on thermal sensitivity of swimming performance (U_crit_) and sarco-endoplasmic reticulum ATPase (SERCA) activity. U_crit_ was determined by an interaction between drug treatment (control, DMSO-only, TSA) and test temperature (Drug x Test), but not between acclimation (**A**: 18 °C, **B**: 28 °C) and drug treatment. TSA (black bars) reduced U_crit_, and this effect was more pronounced at 28 °C. Data from the DMSO-only treatment are shown in Supplementary Fig. S2. TSA decreased SERCA activity in 18 °C-acclimated fish (**C**) but not in 28 °C acclimated (**D**) fish (Drug x Acclimation interaction). SERCA activity was higher at 28 °C than at 18 °C. Means ± s.e. are shown, and N = 8–10 fish per treatment for U_crit_ and N = 6–8 fish for SERCA activity measurements; in case of statistically significant interactions, these are indicated below the title.

Sustained swimming performance (U_crit_) was influenced by an interaction between TSA treatment and acute test temperature (p < 0.037), and TSA reduced U_crit_ to a greater extent at 28 °C compared to 18 °C acute test temperature (Fig. 2A,B). There was no effect of DMSO on U_crit_ (comparison of marginal means, p = 0.24), and results from the DMSO treatment are shown in Supplementary Material Fig. S2. There was a significant interaction between acclimation temperature and test temperature (p = 0.014: Fig. 2A,B), and U_crit_ was higher in the cold acclimated group at the cold test temperature compared to the warm acclimated fish. The interaction between acclimation temperature and TSA treatment was not significant and neither was the three-way interaction (both p > 0.6).

### HDAC increased muscle SERCA activity in cold-acclimated fish but not in warm-acclimated fish

There was a significant interaction between TSA treatment and acclimation temperature (p = 0.0068), and TSA decreased SERCA activity in skeletal muscle of cold-acclimated fish, but not in warm-acclimated fish (Fig. 2C,D). SERCA activity increased with increasing test temperature (p < 0.0001).

### HDAC decreased skeletal muscle slow myosin heavy chain content, which was lower in warm acclimated fish

**Figure 3:**
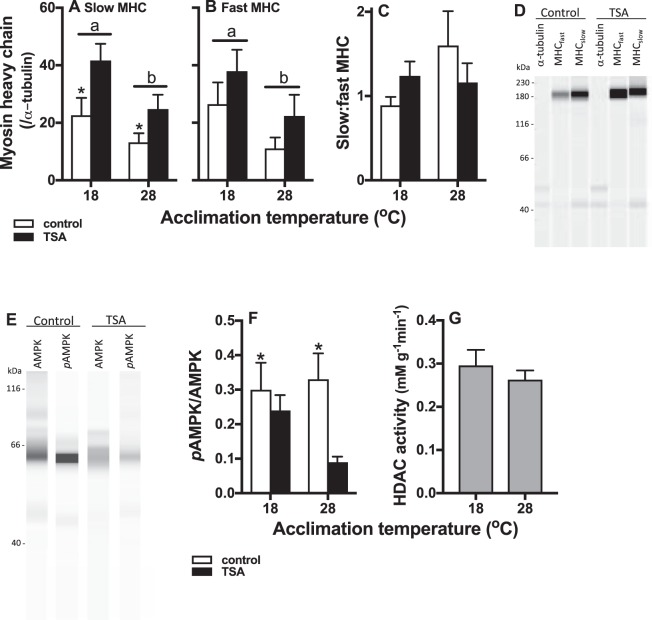
Protein concentrations and HDAC activity in skeletal muscle. Slow (**A**), but not fast (**B**) myosin heavy chains increased significantly following TSA treatment (TSA treatment = black bars, control = open bars). Concentrations of both slow and fast myosin heavy chains were significantly lower in warm acclimated animals. The ratio between slow and fast myosin heave chains (**C**) did not change with any of the treatments. Representative examples of MHC protein bands (and α-tubulin) are shown in (**D**), and of AMPK and *p*AMPK protein bands in (**E**); note however, that protein concentrations were measured by capillary electrophoresis so that the bands in (**D**,**E**) do not represents actual blots, but were generated electronically. AMPK activity (ratio between *p*AMPK:AMPK) decreased following TSA treatment (**F**). There was no difference in total HDAC activity between acclimation treatments (**G**). Means ± s.e. are shown, and N = 6 fish per treatment.

TSA increased slow and fast myosin heavy chain concentrations in skeletal muscle, and this was statistically significant for slow myosin heavy chains (main effect of drug treatment p = 0.014), but not for fast myosin heavy chains (p = 0.13; Fig. 3A,B). Concentrations of both slow and fast myosin heavy chains were lower in warm acclimated animals (main effect of acclimation, p = 0.016 and 0.030, respectively). The interaction between TSA treatment and acclimation was not significantly for concentrations of slow (p = 0.69) or fast (p = 0.98) myosin heavy chains. Similarly, the ratio of slow to fast myosin heavy chains did not change significantly with either acclimation temperature (p = 0.069), TSA treatment (p = 0.54), or their interaction (p = 0.98; Fig. 3C).

### Skeletal muscle AMPK activity was lower in TSA-treated fish, and there was no difference in HDAC activity between acclimation treatments

The ratio between phosphorylated AMPK (*p*AMPK) to total AMPK indicates AMPK activity, which was significantly lower in TSA treated fish (p = 0.016, Fig. 3D). There was no significant effect of acclimation treatment (p = 0.59) or of the interaction between TSA treatment and acclimation (p = 0.15) on AMPK activity. There was no significant difference in HDAC activity between acclimation treatments (p = 0.50; Fig. 3E).

### HDAC activity did not affect heart rate

**Figure 4:**
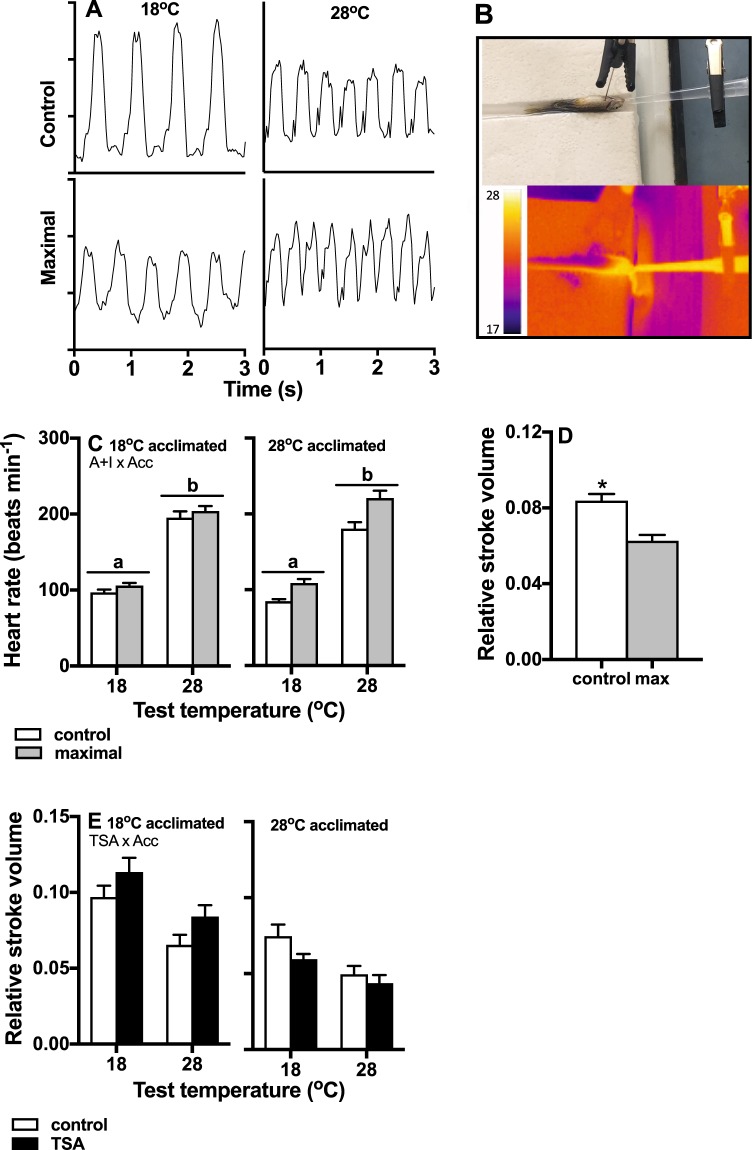
Cardiac responses to TSA, and isoproterenol + atropine treatments which induced maximal heart rates. Example traces showing changes in heart rate and stroke volume with different levels of acute test temperature and A + I treatment (control and maximal) (**A**). The position of the laser Doppler probe is shown in the upper panel (B), and the transition from cool to warm acute test temperature can be seen in the example of a image from the thermal camera (**B**, lower panel; thermal range in photo = 17–28 °C), which we used to monitor acute test temperatures. We used a fine pipette tip inserted into the mouth of the fish to flush the gills and deliver drugs. Heart rate was determined by an interaction between acclimation temperature and A + I, and it increased with increasing acute test temperature (**C**). Relative stroke volume decreased when heart rate was maximal (i.e. with A + I treatment; **D**) and with increasing acclimation and acute test temperatures (**E**). Interestingly, there was a significant interaction between TSA treatment and acclimation temperature, and TSA increased relative stroke volume in cold acclimated fish, but had the opposite effect in warm acclimated fish (**E**). Marginal means (±s.e.) are shown, asterisks indicate significant differences between individual bars, horizontal lines with different letters indicate significant differences between treatment groups, and significant interactions are indicted below the title in individual panels. N = 8–11 fish for each treatment group, and the complete data set is shown in Supplementary Fig. S3.

Example traces show changes in heart rate and stroke volume with different levels of acute test temperature and atropine + isopreteronol (A + I) treatment, which stimulates maximal heart rates (Fig. 4A). The position of the laser Doppler probe is shown in the photo in Fig. 4B (upper panel), and the transition from cool to warm acute test temperature can be seen in the example of a image from the thermal camera, which we used to monitor acute test temperatures (Fig. 4B, lower panel).

Heart rates increased significantly following treatment with atropine + isopreteronol, and this effect was more pronounced in warm-acclimated fish (interaction A + T and acclimation temperature, p = 0.0024). Heart rates were significantly higher at 28 °C acute test temperature compared to 18 °C (p < 0.0001). However, TSA treatment or any of its interactions did not affect heart rates significantly (all p > 0.5; Fig. 4C). Relative stroke volume decreased significantly following A + I treatment (p < 0.0001; Fig. 4D).

### HDAC increased stroke volume in cold-acclimated fish

Relative stroke volume was determined by a significant interaction between TSA treatment and acclimation temperature (p < 0.0001; Fig. 4F). TSA treatment increased stroke volume in cold acclimated fish, but had the opposite effect in warm-acclimated fish (Fig. 4E). Relative stroke volume was significantly lower at the warm acclimation- (p < 0.0001) and acute test temperatures (p < 0.0001) compared to the cold treatments. Analysis of marginal means showed that DMSO-only did not affect either heart rate or stroke volume (p > 0.05) and the complete dataset including DMSO-only treatment is shown in Supplementary Fig. S3.

### HDAC decreased slow and fast MHC concentrations in cardiac muscle

**Figure 5:**
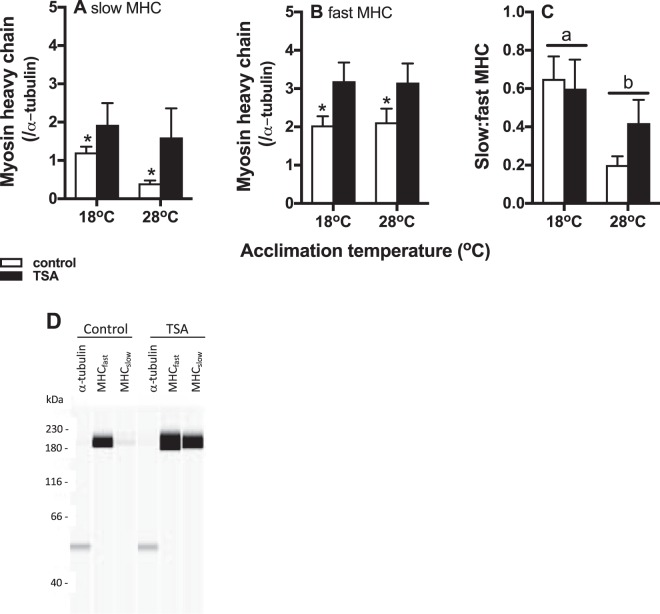
Protein concentrations in cardiac muscle. Slow (**A**) and fast (**B**) myosin heavy chains increased significantly following TSA treatment (TSA treatment = black bars, control = open bars). The ratio between slow and fast myosin heave chains (**C**) was lower in warm acclimated animals. Means ± s.e. are shown, and N = 5–7 fish per treatment. Asterisks indicate significant differences between individual bars, and horizontal lines with different letters indicate significant differences between treatment groups. Representative traces of protein bands are shown in (**D**); note however, that protein concentrations were measured by capillary electrophoresis so that the bands in Fig. 5D do not represents actual blots, but were generated electronically.

There were significant main effects of TSA treatment on MHC concentrations (Fig. 5), and both slow (p = 0.040; Fig. 5A) and fast (p = 0.015; Fig. 5B) MHC concentrations increased following TSA exposure. Acclimation temperature and its interaction with TSA treatment did not affect MHC concentrations significantly (all p > 0.43). The ratio between slow and fast MHC differed significantly between acclimation treatments (p = 0.0068; Fig. 5C) and it was higher in cold acclimated animals; TSA treatment or its interaction with acclimation treatment did not affect MHC ratio (both p > 0.45). Representative protein bands are shown in Fig. 5D.

## Discussion

**Figure 6:**
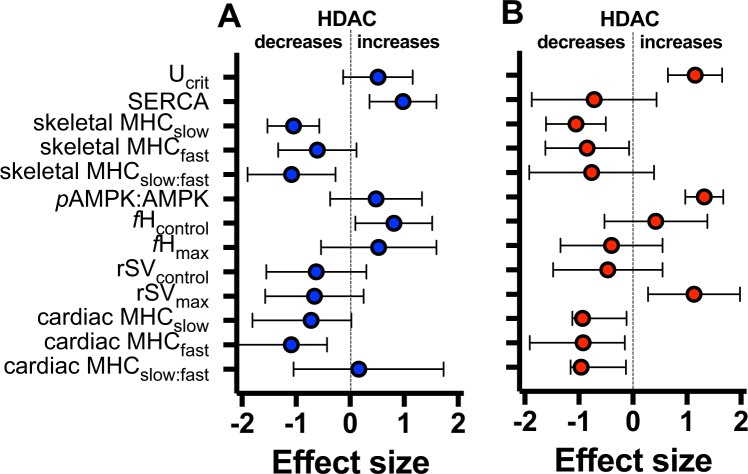
Summary of the effects of HDAC on cold and warm acclimated animals. Effects sizes were calculated as Cohen’s d comparing TSA to control treatments. The effects of HDAC are shown separately for cold acclimated animals when both acute test and acclimation temperatures were 18 °C (**A**) and warm acclimated animals (**B**; both acclimation and acute test temperatures = 28 °C). Effect sizes are shown for the different traits we measured (U_crit_ = sustained swimming performance; SERCA = sarco-endoplasmic reticulum ATPase; MHC = myosin heavy chain; *p*AMPK = activated AMPK; *f*H = heart rate; rSV = relative stroke volume), and error bars show 95% non-parametric bootstrap confidence intervals.

We have shown that HDAC activity alters physiological phenotypes in a temperature-dependent manner. These effects of HDAC ranged from altering metabolomic profiles uniquely in different acclimation treatments to changing the acute thermal sensitivity of locomotor performance. Often, the phenotypic effects of HDAC were specific to different acclimation treatments, and we summarised these as effect sizes of HDAC for cold and warm acclimated animals (Fig. 6). However, the responses were not always as we predicted. We reject the hypothesis that inhibiting HDAC increases locomotor and cardiac function in warm acclimated animals, but we accept the hypothesis that HDAC supresses myosin heavy chain (MHC) content in skeletal and cardiac muscle. We hypothesised that inhibiting HDAC would mimic its export from the nucleus as a result of increased AMPK activity following a decrease in energy status resulting from a decrease in temperature. Reduced AMPK activity following inhibition of HDAC corroborates their interaction, and was to be expected because reduced HDAC activity would restore energy balance by promoting oxidative phenotypes and thereby feed back to reduce AMPK activity^35^. Reduced HDAC activity in warm acclimated animals, however, did not necessarily mimic cold acclimation as we predicted. The significantly different metabolomic profiles of cold and warm acclimated control animals are interesting and novel, and show that chronic exposure to different temperatures leads to differential regulation of metabolic pathways. Inhibiting HDAC did not change the metabolomic profile of warm acclimated animals, but it shifted the metabolomic profile of cold acclimated animals towards that of warm acclimated individuals. Contrary to our expectation, these data indicate that HDAC activity promotes cold acclimation.

Our swimming performance results lead to a similar conclusion. As expected, zebrafish acclimated swimming performance, which manifested as an increase in performance in cold acclimated animals. However, inhibiting HDAC decreased swimming performance. At least for cold acclimated animals, this result may be explained by the reduction in SERCA activity following TSA treatment. SERCA is instrumental for muscle relaxation by sequestering calcium back into the sarcoplasmic reticulum following contraction^36,37^. Increased SERCA mRNA expression was associated with higher sprint and endurance performance in zebrafish^38^, and inhibiting SERCA activity led to reduced fatigue resistance in isolated rat muscle^39^. SERCA activity is determined genomically via regulation of the ATP2a1 and ATP2a2 genes, the mRNA of which are spliced to code for different isoforms of SERCA1 and SERCA 2 proteins, respectively^40,41^. Additionally, SERCA activity is regulated by sarcolipin, phospholamban, and by post-translational modifications such as acetylation of the protein^41,42^. Inhibition of HDAC and subsequent increases in acetylation of SERCA 2a protein increased its activity in cardiac muscle^43^. However, it has also been suggested that increased acetylation may decrease cardiac SERCA2a activity^44^. Additionally, SERCA is inhibited by phospholamban in a calcium-dependent manner where phospholamban is repressed by high intracellular calcium concentrations^42^. Thyroid hormone can promote SERCA1 activity either by enhancing mRNA and protein expression or by suppressing the effect of phospholamban^32,41,45^. Similar to the results for locomotor performance, our results indicate that the effects of acetylation are temperature-dependent in skeletal muscle, and we show that HDAC activity promotes SERCA activity in cold acclimated animals. The effects of HDAC on SERCA may be a combination between deacetylation of the protein directly and genomic effects via histone deacetylation. For example, thyroid hormone, which acts in a temperature-dependent manner^32^, also affects SERCA activity^45^ and interacts with HDAC either directly or via AMPK^46,47^. The next challenge therefore lies in disentangling the interactive effects of different regulatory pathways in mediating thermal plasticity.

In contrast to its effect on SERCA, the effect of HDAC inhibition on myosin heavy chain concentrations was more predictable. Class I and II HDAC suppress the myocyte enhancer factor 2 (MEF2) and thereby also inhibit myosin heavy chain expression, and particular that of slow MHC^33^. We showed that HDAC inhibition led to increased concentrations of MHC in both skeletal and cardiac muscle, although there was no shift towards slow MHC. The MHC results for skeletal muscle are somewhat contradictory to our swimming performance results, because we expected that increased MHC concentrations lead to improved muscle contractile function and thereby improved locomotor performance. It is interesting, however, that muscle of cold acclimated animals had greater content of slow MHC. Sustained locomotor performance may be dependent to a greater extent on slow MHC so that the proportion of slow MHC rather than absolute concentration may explain acclimation of U_crit_, and maybe also of metabolic rates^32^.

Our acclimation data indicate that maximum heart rates are constrained by low acclimation temperature, with limited capacity for compensation for cold temperature. However, relative stroke volume was significantly higher in cold acclimated animals and highest when cold acclimation and acute temperatures coincided. Taken together, these data indicate that there is compensation for cold in cardiac function and that it is mediated primarily by changes in stroke volume in zebrafish. In contrast to skeletal muscle, there was a parallel increase in cardiac muscle MHC content and relative stroke volume of cold acclimated animals. TSA treatment did not affect heart rate, which indicates that rates of muscle contraction and relaxation were not affected by MHC content, but that MHC concentrations affect contractile force and thereby stroke volume^48^.

Calculations of effect sizes (Fig. 6) when acclimation and acute test temperatures coincided indicate that HDAC had a positive effect of heart rates in cold acclimated animals. Hence, at least under those specific circumstances, HDAC promoted cardiac MHC, heart rate, and relative stroke volume, which would lead to overall improved cardiac performance during cold acclimation. The result showing that relative stroke volume was greatest in cold acclimated fish at the cold test temperature in both control and TSA treated fish indicate that there are mechanisms other than HDAC that regulate cardiac thermal plasticity. Again, thyroid hormone is known to promote cold acclimation of cardiac function in zebrafish^29^, and the autonomic nervous system is likely to play a role^49^.

In the experiments reported here, we focussed on the effects of HDAC inhibition on thermal plasticity. However, this treatment should not be interpreted in isolation, and inhibition of HDAC alters the balance between HAT-induced acetylation and deacetylation by HDAC. Ultimately, the balance between these mechanisms determines physiological responses and plasticity^15^. Histone (de)acetylation also does not act in isolation and, as we pointed out above, HDAC interact with other regulatory mechanisms. The interactions between regulatory mechanisms and their evolutionary history therefore determine the capacity for thermal plasticity. The action and evolutionary history of regulators, such as HDAC, AMPK, or thyroid hormone, is often relatively well understood^21,24,50,51^, and this empirically based understanding provides the best predictive power of the efficacy of thermal plasticity across phylogenetic and environmental contexts.

For example, HDAC and AMPK are evolutionarily ancient^21,22^, but other regulatory mechanisms that are known to be important in mediating thermal acclimation have evolved and diversified later in evolutionary history. For example, the actions of thyroid hormone and PGC-1α are relatively derived functions among metazoans, so that plasticity mediated by these regulators must also be a derived trait even if interacting regulators such as HDAC are much more ancient. Hence, the choice of model organisms to study plasticity is crucial, because the regulatory potential to mediate plasticity varies between taxa^51^. Using an invertebrate model, for example, will lack generality because the reduced regulatory potential will constrain selection compared to a vertebrate model. Determining regulatory pathways therefore can make a substantial contribution to predict the importance of plasticity in variable environments.

## Material and Methods

### Study animals and acclimation treatments

All procedures had the approval of the University of Sydney Animal Ethics Committee (approval # 2017/1200). All methods were performed in accordance with the relevant guidelines and regulations. Zebrafish (*Danio rerio*) of mixed sex were obtained from a commercial supplier (Livefish, Bundaberg, QLD, Australia), and fish were kept at 23–24 °C for at least one week before acclimation treatments started. Following this habituation period, we gradually changed the tank temperatures to the acclimation temperatures of 18 °C (cold acclimation) or 28 °C (warm acclimation) over 24 h. Fish were acclimated for three weeks to their respective temperatures, which is sufficient to induce phenotypic responses in this strain of zebrafish^32^. There were 60 fish dispersed across four tanks (645 × 423 × 276 mm) within each acclimation temperature treatment (i.e. eight tanks in total). Each tank contained a filter connected to an air pump (AC-9908; Resun, China), and a 30% water change was done once a week. Acclimation treatments were conducted in a temperature-controlled room set at 18 °C, and higher water temperatures were achieved with submersible heaters (200 W; AquaOne, Kong’s Australia, Ingleburn, Australia). We conducted two identical acclimation treatments subsequent to each other, the first to analyse skeletal muscle responses and locomotion, and the second to analyse cardiac responses; each acclimation treatment followed the same experimental design with 60 fish dispersed across four tanks in for each acclimation temperature (i.e. a total of 16 tanks across both acclimation treatments). No individual fish was used in more than one experiment. Fish were fed twice daily with commercial flake food (Wardley’s Tropical Flakes, The Hartz Mountain Company, Secaucus, NJ, USA) and the light cycle was 12 h dark: 12 h light. Sample sizes for all treatment combinations are given in Supplementary Table S1.

### Trichostatin A treatments

We reduced HDAC activity pharmacologically with the class I and II HDAC inhibitor trichostatin A (TSA)^10,52,53^. After three weeks of acclimation as described above, fish were exposed to one of three treatments (referred to as “TSA treatments” below) in home tank water: (a) control in water only, (b) DMSO (0.2 ml l^−1^), or (c) 500 nM TSA dissolved in DMSO (0.2 ml l^−1^). For the TSA treatments, fish were selected randomly from each of the four replicate tanks within acclimation treatments. TSA treatments were conducted in 1 l containers for 48 h, and fish were fed daily. We have previously determined the efficacy of TSA dissolved in tank water in reducing HDAC activity and increasing histone acetylation within 48 h^34^, and we summarised these results in Supplementary Information Fig. S4. We chose a 48-h period for treatment with TSA, because inhibition of HDAC causes a decrease in mRNA within 2 h^12^, and protein concentrations change within 48 h^34,54^. After the two days of treatment, swimming performance or cardiac responses were measured at 18 °C and 28 °C acute test temperatures. After these trials, fish were euthanised by cervical dislocation, and skeletal muscle tissue or whole hearts were collected for biochemical analyses.

We measured HDAC activity in control fish from each acclimation treatment (N = 6 fish each) using a commercial kit (catalog #17-374, Millipore, Temecula CA, USA) to determine whether acclimation itself affected HDAC activity.

### Metabolomics

We measured global responses to acclimation and TSA treatments (in n = 6 fish per treatment) by determining small metabolite concentrations in skeletal muscle using liquid chromatography - mass spectrometry (LC-MS). We chose metabolomics for these measures because metabolites reflect actual flux through metabolic pathways and therefore closely reflect functional differences. LC-MS analyses were conducted by the Australian Research Facility for Marine Microbial Biotoxins, Sydney Institute of Marine Science, Chowder Bay, Australia on a Q Exactive high resolution mass spectrometer (ThermoScientific, Scoresby, Australia) equipped with an electrospray ionization source. The following source parameters were used in all experiments: a capillary temperature of 256 °C, a spray voltage of 3.5 kV, an auxiliary gas heater temperature of 412 °C, a sheath gas and an auxiliary gas flow rate of 54 and 14 (arbitrary units). The mass spectrometer was operated both in the positive and negative ion mode scanning across the range of m/z 85–1,000. Chromatographic separation was performed on a ACCELA™ UPLC system (Thermo Scientific). Analysis was performed using an Acquity UPLC BEH Amide 130A 1.7 um 150 × 2.1 mm column with a flow rate of 0.3 ml/min and an injection volume of 5 µL. Mobile phase A was 0.01% formic acid in water and B was 0.05% formic acid in acetonitrile. A gradient program with a flow rate of 0.3 ml/min was run starting with a linear gradient from 10% A to 70% A in 12.0 min and then it was held constant for 1.0 min and the flow rate was changed linearly to 0.4 ml/min. The condition was again changed from 70% to 10% A in 2 min while the flow rate was increased linearly to 0.5 ml/min and held for 1 min. The flow rate was then dropped down to the initial condition 0.3 ml/min for 1 min, and was equilibrated another 2 min before sample was injected. Whole muscle samples were homegenised in methanol (1:9).

### Swimming trials

We used independent fish (n = 7–10 fish per treatment) for each acute test temperature, and each fish was swum only once. We determined critical sustained swimming speed (U_crit_)^55^ according to published protocols^56^. U_crit_ was measured in a Blazka-type swimming flume consisting of a clear plastic cylinder (150 mm × 32 mm diameter) tightly fitted over the intake end of a submersible inline pump (12 V DC, iL500, Rule, Hertfordshire, UK). A plastic grid separated the swimming flume from the pump and a bundle of hollow straws at the inlet helped maintain laminar flow; the flume was contained in a plastic tank (645 × 423 × 276 mm). Flow speed was adjusted with a variable DC power supply (NP9615; Manson Engineering Industrial, Hong Kong, China) and flow speed for each flume was measured in real time with a flow meter (DigiFlow 6710 M, Savant Electronics, Taichung, Taiwan). Fish were swum initially for 10 min at 0.1 m s^−1^, when flow velocity in the flume was increased in steps (U_i_) of 0.06 ms^−1^ every 10 min (T_i_). U_crit_ was determined as U_crit_ = U_f_ + T_f_/T_i_ × U_i_, where U_f_ is the highest speed maintained for an entire interval (T_i_), and T_f_ is the time until exhaustion at the final speed interval. A fish was defined to be exhausted when it could no longer keep its position in the water column after two chances; that is, when the fish first fell back on the plastic grid, water flow was reduced immediately for 5–10 s until the fish swam again when flow was increased back to the previous velocity. The next time the fish fell back, the trial was ended. We expressed swimming performance as body lengths per second (BL s^−1^).

### Cardiac performance

We measured cardiac responses with a laser Doppler blood flow meter (ML191, AD Instruments, Sydney, Australia) connected to a PowerLab (ML840, AD Instruments, Sydney, Australia) at 18 °C and 28 °C acute test temperatures (n = 8–11 fish per treatment). Fish were anaesthetized in iso-eugenol (AQUI-S; AQUI-S New Zealand Ltd, Lower Hutt, New Zealand), with 0.040 µl per 1 ml of water to induce anaesthesia and 0.028 µl ml^−1^ to maintain anaesthesia. We used AQUI-S rather than tricaine melthanesulphonate (MS222) because it does not interfere with vagal nerve transmission or elevate resting cortisol levels, both of which could affect stroke volume and heart rate^57,58^. Anaesthesia was maintained by pumping AQUI-S solution continuously through the oral cavity of the fish and across its gills via a small pipette tip connected to a peristaltic pump (i150, iPumps, Tewekesbury, UK) by rubber hosing. Fish were placed ventral side up into a groove cut into a rectangular foam platform. The groove was cut to hold the fish comfortably in place (approximately 4 mm wide at the top and 60 mm long), and the peristaltic flow kept the surface of the fish moist. An OxyFlo Needle Probe (MNP110XP, Oxford Optronix Ltd, Milton, Abingdon, UK) was positioned with a stereotaxic apparatus so that it touched the ventral surface of the fish, just anterior to the heart^29^. Once a clear signal of flow was established, neither the fish nor the probe was disturbed until measurements at both temperatures were complete (see below). The signals were sampled at 40 Hz by Chart software (AD Instruments).

We elicited maximum heart rates by administering atropine (20 µM; Sigma Aldrich, Castle Hill, Australia) to block cholinergic receptors plus isoproterenol (20 µM; Sigma Aldrich, Castle Hill, Australia) to stimulate ß-adrenergic receptors^59,60^; we refer to this drug treatment as A + I treatment, which elicited “maximal” heart rates. Atropine and isoproterenol were delivered to the gills together with the anaesthetic, and measurements were taken with (maximal) and without (control) drugs at the two different test temperatures. We maintained separate flasks containing either anaesthetic only or anaesthetic plus drugs in water baths at 16 °C and 30 °C, and we changed the peristaltic pump inlet between flasks to achieve the appropriate treatments. Fish temperatures were monitored with a thermal imaging camera (model 875, Testo, Lenzkirch, Germany), and measurements were recorded after fish had reached thermal equilibrium; water baths were at slightly more extreme temperatures than the desired 18 °C and 28 °C treatment temperatures to account for heat exchange in the rubber tubing. The sequence of treatments within each fish was: (i) anaesthetic-only at test temperature 1 (5 min of exposure to treatment conditions before measurements), where the first test temperature was alternated between replicate fish, (ii) anaesthetic-only at test temperature 2 (5 min), (iii) anaesthetic plus atropine/isoproterenol at test temperature 2 (10 min), and (iv) anaesthetic plus atropine/isoproterenol at test temperature 1 (5 min). We monitored cardiac responses for 10 min during the first atropine/isoproterenol exposure to ensure that the drugs had taken effect^59^. Heart rates could be discerned easily from the spikes in the flow rate, and we calculated relative stroke volume as the integral of flow above baseline during a single heartbeat^29^. In the analysis, we used the average of heart rate and stroke volume of three periods of 10 beats each for each treatment condition per fish. Measurements of relative flow can differ between fish because of slight differences in needle probe positioning. We therefore normalised stroke volume measurements between individual fish within treatments groups by subtracting residuals.

### SERCA activity

SERCA assays (n = 6 fish per treatment) were conducted according to published protocols^34,39^. Briefly, muscle samples were homogenized (in a TissueLyser LT; Qiagen, Venlo, The Netherlands) in nine parts homogenization buffer (250 mM sucrose, 5 mM EDTA, and 20 mM imidazole, pH 7.2). Under constant vortexing, 0.1% sodium deoxycholate dissolved in homogenization buffer was added to muscle tissue homogenate in equivalent amounts (w/v). Sodium deoxycholate-treated homogenates were preincubated for 10 min in assay medium (25 mM imidazole, 0.2 mM, CaCl_2_, 80 mM KCl, 5 mM MgCl_2_) with and without 10 M of thapsigargin, a specific inhibitor of SERCA^61^. The activity of SERCA was determined in duplicate assays using a UV/visible spectrophotometer (Ultrospec 2100 Pro; GE Healthcare, Silverwater, Australia). SERCA activity was measured by quantifying the liberation of inorganic phosphate. The assay was initiated by the addition of 3 mM ATP to homogenates, left to incubate for 5 min, and the reaction was stopped by the addition of 0.4 M perchloric acid. The final solution was centrifuged (1,200 g, 15 min at 4 °C) and the supernatant was added to 1 part colour reagent (8 mM ammonium molybdate, 335 mM concentrated H_2_SO_4_, 145 mM FeSO_4_) to quantify the amount of inorganic phosphate relative to a standard curve; the standard curve was determined with known concentrations of PO_4_ (25–250 nM). After 10 min of colour formation absorbance was read at 750 nm. SERCA activity (mol of product min^−1^ g^−1^ of wet tissue) was calculated as the difference in inorganic phosphate liberated in the presence and absence of thapsigargin. Assays were conducted at 18 °C and at 28 °C in samples from N = 6 fish for each combination of drug treatment (TSA and control), acclimation- and test temperature. We did not determine SERCA activity in DMSO-only treated fish because DMSO alone did not have an effect on swimming or cardiac performance (see Results).

### Protein concentrations

We determined protein concentrations (n = 5–7 fish per treatment) by capillary electrophoresis in a Wes Simple Western System (Protein Simple, San Jose, CA, USA) according to the manufacturer’s instructions. We measured protein concentrations in skeletal and cardiac muscle of control and TSA-treated fish from each acclimation treatment. As for SERCA, we did not measure protein concentrations in DMSO-only treated fish. Samples used for measurements of protein concentrations were homogenized (in a TissueLyser LT; Qiagen, Venlo, The Netherlands) in 9 vol RIPA buffer (20 mM Tris·HCl, pH 7.5, 150 mM NaCl, 1 mM EDTA, 1 mM EGTA, 1% NP40, 1% sodium deoxycholate) and protease and phosphatase inhibitor cocktail (Roche Life Sciences, Germany) solution. Antibodies we used were EB165 (skeletal muscle fast MHC; Developmental Studies Hybridoma Bank, University of Iowa, Iowa City, IA, USA), BA-F8 (skeletal muscle slow MHC; Developmental Studies Hybridoma Bank, University of Iowa), S46 (cardiac slow MHC; Developmental Studies Hybridoma Bank, University of Iowa), F59 (cardiac fast MHC; Developmental Studies Hybridoma Bank, University of Iowa), C5B11 (acetyl-H3K9; Cell Signaling, Danvers, MA, USA), ab80039 (total AMPK; Abcam, Cambridge, MA, USA), ab133448 (phosphorylated AMPK; Abcam, Cambridge, MA, USA), and 12G10 (α-tubulin; Developmental Studies Hybridoma Bank, University of Iowa) as internal control. Before protein assays, the concentrations of protein extracts were determined using a bicinchoninic acid assay kit (Sigma-Aldrich, Castle Hill, Australia) following manufacturer’s instructions.

### Statistical analysis

We analysed U_crit_, cardiac responses, HDAC activity, and protein concentrations with permutational analyses in the package lmPerm^62^ in R^63^. Permutational analyses do not make assumptions about underlying data distributions, but use the data per se to infer significant differences, which is preferable especially for sample sizes that are small relative to the total population of all possible samples^64^. The p-value in permutational tests has the practical meaning of denoting the number of randomized permuted datasets for which the results were as, or more extreme than the observed experimental data divided by the total number of permutations^65^. Hence, p-values in permutational analyses are not associated with any particular distribution, and there are no test statistics(such as F or t). We analysed U_crit_ (in body lengths s^−1^) and protein data with TSA treatment (control, DMSO only, and 500 nM TSA), acclimation temperature (18 and 28 °C) and test temperature (18 and 28 °C) as independent factors. Where appropriate, we used pair-wise permutational tests for post hoc comparisons of marginal means. We analysed heart rates and relative stroke volume with TSA treatment, acclimation temperature, acute test temperature, and cardiac drug treatment (control or atropine + isoproterenol) as factors. We used individual fish ID as a random factor in the analyses of cardiac responses because we took repeated measurements on the same fish.

Metabolomics data were processed initially in proprietorial Sieve software (Thermofisher Scientific, USA), to normalise peaks against blank readings. We analysed mass peaks in MetaboAnalyst 3^66^. Data were pre-treated by median normalisation, cube-root transformation, and Pareto scaling^67^. We conducted partial least squares discriminant analysis^68^ to determine separation of the experimental groups, using 95% confidence intervals for comparisons. We used Significance Analysis of Microarrays (SAM)^69^ to determine the number of metabolites that were significantly differently expressed between treatments at a false discovery rate (FDR) of 0.049 and p < 0.05.

Effect sizes of the TSA treatment on different responses were calculated as Cohen’s d^70^, to compare TSA treatment and control within each acclimation treatment at the test temperature that coincided with the acclimation temperature. We determined 95% confidence intervals for effect sizes by bootstrapping in the package boot in R^63^. All sample sizes are summarised in Supplementary Table S1.

## Supplementary information

Supplementary material

Supplementary data set 1

Supplementary data set 2

## Data Availability

Data are attached as Supplementary Information.
