# Supplementary material for: Histone deacetylase activity mediates thermal plasticity in zebrafish (Danio rerio)
Source: Sci Rep. 2019 Jun 3;9:8216. doi: 10.1038/s41598-019-44726-x (PMC6546753; doi:10.1038/s41598-019-44726-x)
Supplement: Supplementary file 1 — Supplementary material [file 41598_2019_44726_MOESM1_ESM.docx]

**Histone deacetylase activity mediates thermal plasticity in zebrafish (*Danio rerio*)**

Frank Seebacher^1*^ and Alec I. M. Simmonds^1^

*^1^School of Life and Environmental Sciences, The University of Sydney, NSW 2006, Australia.*

**Supplementary Information**

*TSA dose response*

We conducted experiments with zebrafish previously (Simmonds and Seebacher 2017) to determine the efficacy of delivering trichostatin A (TSA) dissolved in tank water. We conducted three treatments (N = 9 fish each): a) TSA dissolved in DMSO (0.2 ml l^-1^) to give a final concentration of 100 nM TSA in tank water; b) 500 nM concentration of TSA in tank water, and c) DMSO dissolved in tank water at the same concentration as for a) and b) above (0.2 ml l^-1^).

After two days in the TSA treatments, fish were euthanised and muscle tissue was dissected to determine activity of type I and II histone deacetylases (HDAC). We determined HDAC activity with a commercial kit (catalog #17-374, Millipore, Temecula CA, USA) following the manufacturer's instructions. There were significant differences in HDAC activity between TSA dose treatments (permutational analysis [see methods in main text] p < 0.05, and HDAC activity was significantly lower in the 500 nM TSA treatment compared to the control (DMSO-only) treatment (p < 0.006; Fig. S1), but the control treatment was not significantly different from the 100 nM TSA treatment (p = 0.10). Exposure of fish to 500 nM TSA for 48 h resulted in significantly increased protein levels (determined by capillary electrophoresis, see main text) of acetylated histone 3 (H3K9; Fig. S1). Hence, in the experiments we exposed fish for two days to 500 nM TSA in tank water to test the effect of histone acetylation on acclimation of swimming performance.

**Figure captions**

**Fig. S1** Summary of the hypotheses tested. We tested whether inhibition of class I and II HDACs with TSA, which would mimic its removal from the nucleus, induces cold-acclimation responses (1 in Figure). As a corollary, we expected AMPK activity (*p*AMPK) to decrease with TSA treatment (2) as a result of feedback from reduced HDAC activity, which would restore energy balance by promoting more oxidative phenotypes. We predicted that inhibition of HDAC is accompanied by an increase in myosin heavy chain (MHC) content, and a shift from fast to slow myosin heavy chain (MHC) isoforms in skeletal and heart muscle (3), as a result of increased myocyte enhancer factor 2 (MEF2) activity. Together, these changes were predicted to increases sustained locomotor performance and cardiac function in warm-acclimated animals (4).

**Fig. S2** Complete dataset of swimming performance. There was an interaction between treatment (control, DMSO-only, TSA) and test temperature, but not between acclimation (18oC left panel, 28oC right panel) and drug treatment. TSA (black bars) reduced swimming performance (U_crit_) and this effect was more pronounced at high test temperature (28^o^C). There were no differences between the control (open bars) and DMSO-only (grey bars) treated fish. Means ± s.e. are shown, and N = 8-10 fish per treatment.

**Fig. S3** Cardiac responses to TSA and atropine + isoproterenol treatments. Heart rates (left panels) were not affected by TSA treatments (control, DMSO-only, and TSA). There was an interaction between acclimation temperature (blue bars = cold acclimated; red bars = warm acclimated) and A+I treatment (dark shading = control; light shading = atropine + isoproterenol) in determining heart rates, and heart rates increased with increasing acute test temperature. Relative stroke volume (right panels) was determined by an interaction between TSA treatment and acclimation temperatures, and stroke volume decreased with increasing acclimation and acute test temperatures.

**Fig. S4** Preliminary experiments testing the efficacy of the TSA treatment. Compared to control treatments, 500 nM of the HDAC inhibitor Trichostatin A (TSA) significantly reduced HDAC activity (A). Inhibition of HDAC with 500 nM TSA led to significantly increased histone 3 (H3K9) acetylation (B). Means ± s.e. are shown (N = 6 per treatment group) and significant differences are indicated by an asterisk. Data redrawn from Simmonds and Seebacher (2017).


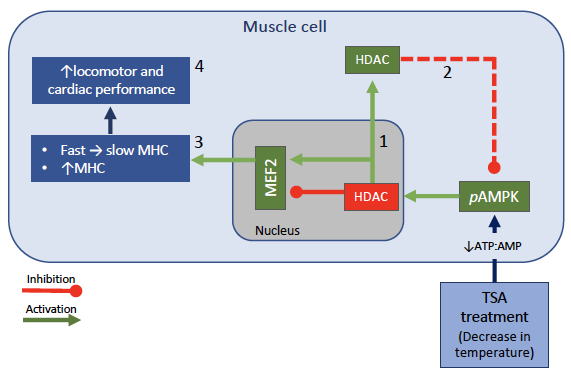


**Figure S1**

**

**Figure S2**

**

**Figure S3**

**Figure S4**

| Treatments | | | Phenotypic responses | | | | | | | |
| --- | --- | --- | --- | --- | --- | --- | --- | --- | --- | --- |
| TSA | Accl. | Test temp. | U_crit_ | HDAC activity | SERCA | Skeletal muscle proteins^a^ | *f*H^b^ | rSV^b^ | Cardiac muscle proteins^a^ | Metabolomic  analysis^a^ |
| control | 18 | 18 | 8 | 6 | 6 | 6 | 11 | 11(10) | 6 | 6 |
| control | 18 | 28 | 10 |  | 6 |  | 11 | 11 |  |  |
| control | 28 | 18 | 8 | 6 | 6 | 6 | 8 | 8 | 7 | 6 |
| control | 28 | 28 | 8 |  | 6 |  | 8 | 8 |  |  |
| DMSO | 18 | 18 | 10 |  |  |  | 9 | 9 |  |  |
| DMSO | 18 | 28 | 9 |  |  |  | 9 | 9 |  |  |
| DMSO | 28 | 18 | 8 |  |  |  | 8(6) | 8(5) |  |  |
| DMSO | 28 | 28 | 9 |  |  |  | 8(7) | 8(5) |  |  |
| TSA | 18 | 18 | 10 |  | 6 | 6 | 11 | 11(10) | 5 | 6 |
| TSA | 18 | 28 | 10 |  | 6 |  | 11 | 11(10) |  |  |
| TSA | 28 | 18 | 7 |  | 6 | 6 | 9 | 9 | 5 | 6 |
| TSA | 28 | 28 | 8 |  | 6 |  | 9 | 9 |  |  |

**Table S1** Sample sizes (numbers of fish) for different treatments (TSA = trichostatin a treatment [control, DMSO only, TSA]; Accl. = thermal acclimation [18 and 28^o^C]; Test temp. = acute test temperature [18 and 28^o^C] ) and phenotypic response measures (U_crit_ = sustained swimmign speed; SERCA = sarcoendoplasmic reticulum Ca^2+^-ATPase; fH = heart rate; rSV = relative stroke volume)

^a^note that Test Temperature is not a factor for this measurements

^b^numbers in parentheses refer to maximal heart rate or stroke volume if these were different from resting values
